# Antioxidants in Potatoes: A Functional View on One of the Major Food Crops Worldwide

**DOI:** 10.3390/molecules26092446

**Published:** 2021-04-22

**Authors:** Hanjo Hellmann, Aymeric Goyer, Duroy A. Navarre

**Affiliations:** 1 School of Biological Sciences, Washington State University, Pullman, WA 99164, USA; 2 Hermiston Agricultural Research and Extension Center, Department of Botany and Plant Pathology, Oregon State University, Hermiston, OR 97838, USA; aymeric.goyer@oregonstate.edu; 3 USDA-ARS, Prosser, WA 99350, USA; roy.navarre@usda.gov

**Keywords:** potato, antioxidant, vitamin, glycoalkaloids, patatin, phenolic antioxidants, nutrition, health, climate change

## Abstract

With a growing world population, accelerating climate changes, and limited arable land, it is critical to focus on plant-based resources for sustainable food production. In addition, plants are a cornucopia for secondary metabolites, of which many have robust antioxidative capacities and are beneficial for human health. Potato is one of the major food crops worldwide, and is recognized by the United Nations as an excellent food source for an increasing world population. Potato tubers are rich in a plethora of antioxidants with an array of health-promoting effects. This review article provides a detailed overview about the biosynthesis, chemical and health-promoting properties of the most abundant antioxidants in potato tubers, including several vitamins, carotenoids and phenylpropanoids. The dietary contribution of diverse commercial and primitive cultivars are detailed and document that potato contributes much more than just complex carbohydrates to the diet. Finally, the review provides insights into the current and future potential of potato-based systems as tools and resources for healthy and sustainable food production.

## 1. Introduction

Plants have the amazing capability to utilize comparably simple ingredients, such as CO_2_, sunlight, and soil-based nutrients, for synthesis of highly complex, organic molecules that serve as building blocks for general development and cellular processes. Compared to more mobile organisms, the sessile lifestyle of plants forces them to deal with any changes in their local environment, and plants have thus developed a complex and powerful array of secondary metabolites to actively respond.

Humans depend on plants as primary food resources, not only to gain access to the basic carbohydrates and amino acids, but also for a variety of metabolites that we lack the ability to synthesize. Plants make an array of organic molecules with crucial, beneficial impacts on human physiology, such as numerous antioxidants, which are molecules that can inhibit oxidation processes. In principle, this is mostly related to the presence of reactive oxygen species (ROS) that can quickly undergo redox reactions in the cell, and thereby significantly alter and damage other metabolites, DNA, proteins or fatty acids [1,2].

Common ROS are, for example, hydroxyl radicals (OH·), superoxide anions (O_2_^−^), singlet oxygen (^1^O_2_), or hydrogen peroxide (H_2_O_2_), and they are generally present in any part of a cell over the course of a day [3,4,5,6]. However, they often rise significantly under stressful conditions, such as abiotic stress (e.g., heat, drought or high light) or high metabolic activities, and can then be extremely harmful to the cell [6]. In such cases it is crucial for cells to have effective antioxidative buffering systems in place that quickly detoxify ROS. Some of the classical non-enzymatic antioxidants are vitamins like vitamin C or E, but similarly flavonoids and other phenolic compounds are also powerful antioxidants [7]. In addition, plant peptides and whole proteins have been recognized in recent years to possess antioxidative capacities [8]. As stated above, most of these antioxidants and other secondary metabolites are not de novo synthesized in humans and need to be provided in the diet, mainly through plant-based resources, to provide beneficial impacts.

We currently face unprecedented challenges with the growing world population, ongoing climate changes, and continuing loss of arable land [9,10,11]. A plant-based diet is widely recognized as a healthier and more sustainable alternative to diets that rely heavily on meat [12,13,14,15]. This can be due to basic nutritional reasons, such as reducing risk of cardiovascular diseases, diabetes or obesity, or for environmental reasons like preserving the CO_2_ balance, and conserving arable land use [12,16]. It is therefore imperative to advocate for research and development of plants for optimal consumption value and agriculture yields to face current challenges and bolster food security for future generations.

Potato (*Solanum tuberosum*) has been identified by the Food and Agriculture Organization of the United Nations (FAO) as a staple and sustainable food for the growing world population (http://www.fao.org/potato-2008/en/aboutiyp/index.html, accessed on 21 April 2021) [17]. Unfortunately, potato has sometimes a negative image, especially in industrialized countries, as a rather unhealthy fast-food resource, mainly due to the consumption of French fries and chips, which does not give this crop its appropriate credits. Besides being a staple food that provides a high-calorie-based, nutrient-rich diet, it is more conservative in land and water usage compared to other major crops, such as wheat or rice [18]. Importantly, the potato tuber does not simply consist of starch, but as discussed below actually has a plethora of secondary metabolites and storage proteins that can have beneficial impacts on the human diet and health.

In this review, we provide detailed insights on the phytonutrient content of the potato tuber with a major focus on antioxidants, their biosynthetic pathways, benefits for human physiology, and potential medical applications. Where applicable we will discuss approaches and potentials of changing the content of antioxidants. Overall, this work is meant to provide an innovative view on potato to emphasize that it is much more than a fast-food resource, but actually an ideal crop to deliver good nutrition and provide agricultural solutions to some of the great challenges we currently face.

## 2. Vitamin-Based Antioxidants

### 2.1. Vitamin B_1_ (Thiamin)

**Figure 1:**
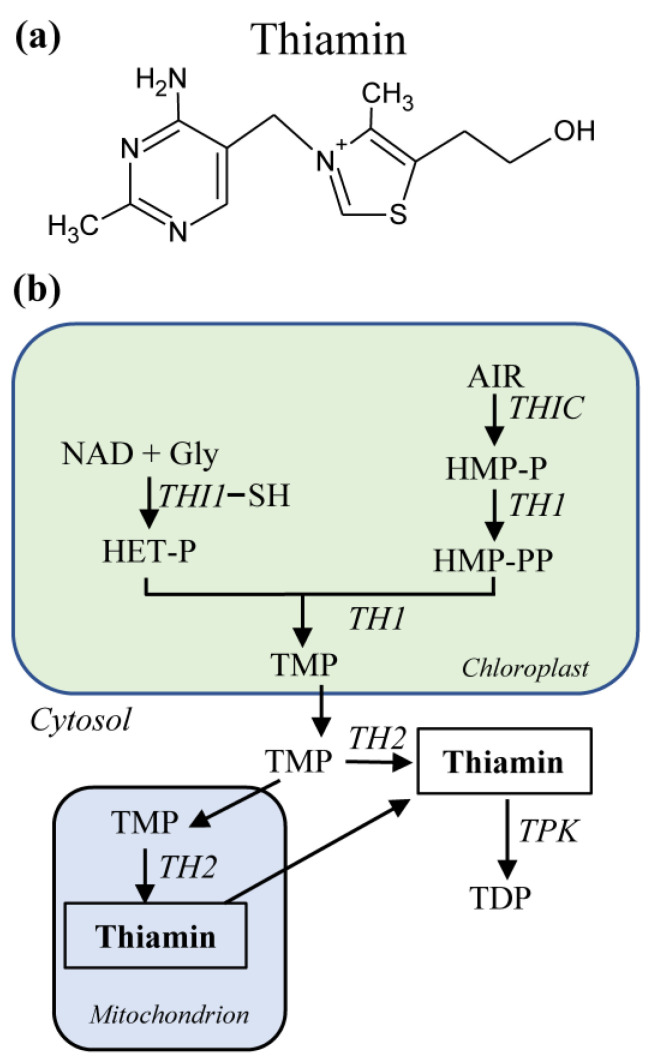
Thiamin biosynthesis in higher plants. (**a**) Chemical structure of thiamin. (**b**) Thiamin biosynthesis requires steps in the chloroplast, cytosol, and the mitochondria. AIR, 5-aminoimidazole ribonucleotide; Gly, glycine; HET-P, 4-methyl-5-β-hydroxyethylthiazole phosphate; HMP-P, 4-amino-2-methyl-5-hydroxymethylpyrimidine phosphate; HMP-PP, 4-amino-2-methyl-5-hydroxymethylpyrimidine diphosphate; TH1, HMP-P kinase/TMP pyrophosphorylase; THI1, HET-P synthase; TH2, TMP phosphatase; THIC, HMP-P synthase; TMP, thiamin monophosphate; TPK, thiamin pyrophosphokinase.

Thiamin is a bipartite molecule made of a pyrimidine (4-amino-2-methyl-5-pyrimidyl) ring linked to a thiazole (4-methyl-5-β-hydroxyethylthiazolium) ring by a methylene bridge (Figure 1a). Thiamin can be phosphorylated to its hydroxyl group to form thiamin mono-, di-, or tri-phosphates. Another form of thiamin, adenosine thiamin triphosphate, has been detected in small amounts in yeast, animal and plant tissues, and its levels increased substantially in carbon-starved *Escherichia coli* [19].

Thiamin is best known for its role as an enzymatic cofactor in its diphosphate form (ThDP) for various important enzymes such as transketolase in the oxidative pentose phosphate pathway, α-ketoglutarate dehydrogenase in the Krebs cycle, and acetolactate synthase in the synthesis of branched-chain amino acids leucine, isoleucine and valine [20,21]. As such, thiamin is essential for growth and development, and for the proper functioning of the heart, muscles and nervous system [22,23,24,25]. However, there is growing evidence that thiamin may also play a role as an antioxidant, either directly or indirectly. Thiamin’s direct antioxidant activity is substantiated by several in vitro and in vivo studies. For instance, Gliszczynska-Swiglo [26] showed that micromolar concentrations of thiamin had antioxidant activities in the trolox equivalent antioxidant capacity (TEAC) assay with ABTS˙^+^ radical cation (2,2′-azinobis (3-ethylbenzothiazoline-6-sulfonic acid)), and that thiamin antioxidant activity was only ~3 times lower than that of ascorbic acid. Lukienko et al. [27] showed that micromolar concentrations of thiamin could inhibit oleic acid peroxidation in vitro. Okai et al. [28] showed that thiamin and thiamin diphosphate have radical scavenging activities against superoxide and hydroperoxide in cell-free systems, and against oxygen radicals in a cellular system. Micromolar concentrations of thiamin could inhibit lipid peroxidation in rat liver microsomes as determined by the decreased accumulation of malonic dialdehyde [27]. In addition, thiamin could prevent oxidation of SH group in cells [29]. Thiamin also prevented growth inhibition induced by paraquat in *E. coli*, and inhibited the induction of antioxidant genes, suggesting a direct antioxidant activity of thiamin against the superoxide anion produced by paraquat treatment [30]. Similarly, thiamin supplementation in growth media prevented root growth inhibition induced by paraquat in Arabidopsis [31].

Thiamin may also have an indirect antioxidant activity. Indeed, thiamin (1–6 mM) attenuated the inhibition of the thiamin-dependent pyruvate dehydrogenase and α-ketoglutarate dehydrogenase by copper-induced radicals [32,33]. Thiamin could prevent cell death induced by carbonyl stress in rat cell systems, probably by preventing the inhibition of pyruvate dehydrogenase and α-ketoglutarate dehydrogenase [33]. Thiamin could protect mouse neuroblastoma cells against paraquat-induced oxidative stress, likely via a role in antioxidant signaling pathway [34]. Finally, thiamin diphosphate plays a major role in the production of reducing power in the form of NADPH as a cofactor of transketolase in the oxidative pentose phosphate pathway [20]. Thiamin antioxidant activity may also work in concert with other antioxidants. For instance, Wang et al. [35] showed that a combination of thiamin and vitamin C can lessen damages to liver cells caused by lead-mediated oxidative stress in mice.

Although humans can synthesize the enzymatic cofactor thiamin diphosphate from thiamin [36], they cannot synthesize thiamin de novo and must obtain it from food, a major source being plants. Plants synthesize thiamin monophosphate (TMP) in the chloroplast by condensing 4-amino-2-methyl-5-hydroxymethylpyrimidine diphosphate (HMP-PP) and 4-methyl-5-β-hydroxyethylthiazole phosphate (HET-P) via the bifunctional HMP-P kinase/TMP pyrophosphorylase TH1 [37] (Figure 1b). HMP-P is produced from 5-aminoimidazole ribonucleotide (AIR) by HMP-P synthase (THIC), a reaction that requires *S*-adenosylmethionine (SAM) and reduced nicotinamide [38]. HMP-P is then phosphorylated to HMP-PP by TH1. HET-P is formed from NAD and glycine by HET-P synthase (THI1), which provides the necessary sulfur from an active-site cysteine, and is thus a single-turnover reaction [39]. True catalytic THI1 paralogs that do not contain an active-site cysteine but use sulfide as sulfur donor have recently been identified in some cereals [39]. TMP is then dephosphorylated to thiamin by TMP phosphatase TH2 in the cytosol or mitochondria [40,41]. Thiamin is then pyrophosphorylated to TDP by thiamin pyrophosphokinase (TPK) in the cytosol [42].

The RDA for thiamin is 1.2 mg/day for men and 1.1 mg/day for women. Nutrition facts on labels of potato bags in retail stores indicate that a medium size potato (148 g) provides 8% of the RDA. However, thiamin content can vary greatly between potato genotypes, with reported concentrations ranging from 292 to 2325 ng g^−1^ FW [43,44]. Some wild species of potato could contain up to two-fold the amount of thiamin found in potato varieties commonly grown in the United States [44], and could be used as gene sources for breeding thiamin-enriched varieties. Genetic engineering of the thiamin biosynthesis pathway is another biofortification alternative that has shown promise in rice endosperm [45] and should be tested in potato tubers. Environmental growth conditions can also affect thiamin content. For instance, total thiamin content in maize seedlings increased ~55% under water and salt stress, and 200% under oxidative stress [46]. However, to our knowledge, there is no data available in potato in response to stress, except a report by Goyer and Haynes [43] that showed no effect of cold temperature on thiamin content in potato tubers.

### 2.2. Vitamin B_6_ (Pyridoxine)

**Figure 2:**
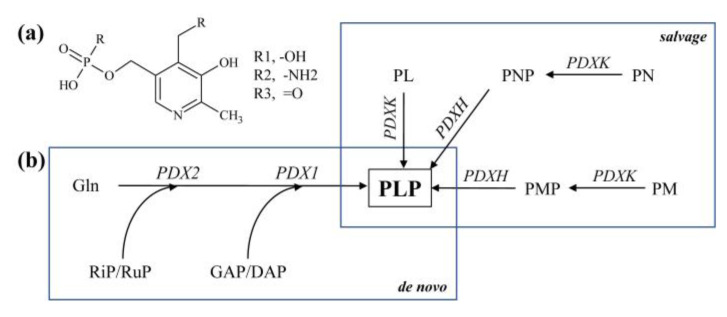
Vitamin B_6_ biosynthesis in higher plants. (**a**) Chemical structure of phosphorylated B_6_ vitamers. (**b**) Vitamin B_6_ de novo biosynthesis and *salvage* pathways. Both pathways are present in the cytosol. Because it is unclear whether they are also active in the chloroplasts, we did not assign a specific cellular localization for either pathway. Either RiB or RuP, as well as GAP or DAP, can be used. phosphate; RuP, ribulose 5′-phosphate; RiP, ribose 5′-phosphate; DXP, deoxyxylulose 5′-phosphate; Gln, glutamine; PL, pyridoxal; PLP, pyridoxal 5′-phosphate; PM, pyridoxamine; PMP, pyridoxamine 5′-phosphate; PN, pyridoxine; PNP, pyridoxine 5′-phosphate; PDXK, pyridoxine kinase; PDXH, pyridoxine dehydrogenase.

Vitamin B_6_ comprises a group of six isomers that share a 5-hydroxy-3,6-methylpyridin ring. They mainly differ in their carbon 4 position where either an aldehyde (pyridoxal (PL)), an amino methyl group (pyridoxamine (PM)), or a hydroxyl methyl group (pyridoxine (PN)) can be present [47] (Figure 2). In addition, all three isomers can be phosphorylated at the methyl group present at the carbon 3 site, leading to pyridoxal-5-phosphate (PLP), PMP, and PNP, respectively, with PLP being the major bioactive form [47]. The phosphorylation is required for the vitamin to function as a co-factor in a broad range of deamination, racemization, and decarboxylation reactions, mainly connected with amino acid metabolism [48]. However, PLP is also required in starch and glycogen degradation, as well as in the biosynthesis of plant hormones, chlorophyll, and certain neurotransmitters, and fatty acid metabolism [48,49,50,51,52,53,54,55]. In addition, it has been demonstrated that the vitamin has strong antioxidative capabilities that can quench a variety of ROS including, for example, OH· or ^1^O_2_ [56,57,58,59]. Besides its requirement as a cofactor in enzymatic reactions, vitB_6_ improves plant tolerance to abiotic factors like high light, UV, or osmotic stress [58,60,61,62], while in humans a broad range of health benefits with positive impacts against anemia, neurological disorders, premenstrual syndrome, cardiovascular diseases, and cancer have been described [63,64,65,66,67,68]. Broad vitB_6_ deficiencies have been documented, for example, in the United States [69], in elderly people in Norway [70], or for young adult women in Canada [71], prompting fortification of food with this vitamin for certain populations.

Plants synthesize PLP de novo using two different enzymes called Pyridoxine Biosynthesis 1 (PDX1) and PDX2 [72] (Figure 2b). In addition, they have a *salvage* pathway, which generates PLP from any of the other five B_6_ vitamers [73]. Humans lack a de novo biosynthesis pathway, but they have the required *salvage* pathway enzymes [74,75]. The vitamin is therefore essential for the human diet, and any of the six B_6_ vitamers can be used as a PLP resource.

The RDA values for vitB_6_ are 1.3 mg for adults (19–51 years), but for males and females above 51 years, slightly higher values of 1.7 mg and 1.5 mg, respectively, are recommended [76]. Potato is a very good source for the vitamin since 100 g of a raw, white-fleshed potato already provide around 12% of the RDA value or 0.239 mg (USDA National Nutrient Database for Standard Reference, Release 17). Processed potato products with reduced water content have higher values, with baked potatoes reaching 0.301 mg 100 g^−1^ and potato chips 0.78 mg/100 g^−1^, which is nearly 50% of the RDA values. In addition, the vitB_6_ content can vary significantly between different potato varieties (e.g., white versus red fleshed) and age of the tubers when sold [77,78].

Because the de novo biosynthetic pathway of PLP is a comparably simple catalyzation by two enzymes, genetic engineering appears to be a feasible way to enhance vitB_6_ levels in the plant. In fact, several studies in Arabidopsis, rice and potato demonstrated that overexpression of PDX proteins can increase vitB_6_ content [61,79,80]. In potato overexpression of a *PDX2* gene from Arabidopsis resulted in enhanced vitB_6_ levels of up to 150% compared to wild type plants [61]. In addition, the plants showed higher tolerance to salt stress (induced by NaCl) and ROS (caused by methyl viologen) [61]. These data show that vitB_6_ biofortification of the tuber may not only increase tuber’s nutritional value but also benefit the plant via vitB_6_ antioxidant activities.

### 2.3. Vitamin B_9_ (Folate)

**Figure 3:**
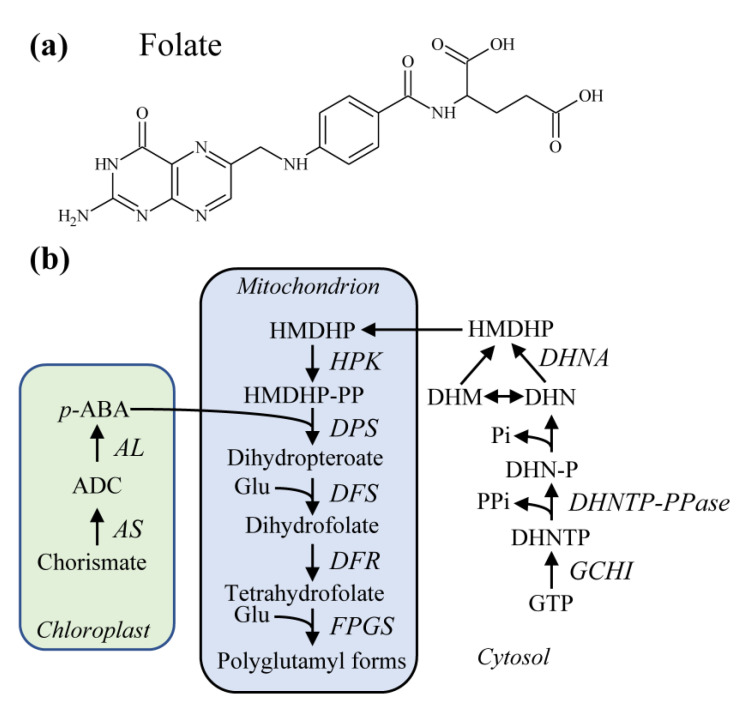
Folate biosynthesis in higher plants. (**a**) Chemical structure of folate. (**b**) The biosynthesis involves chloroplastidic, mitochondrial, and cytosolic steps. *p*-ABA, *p*-aminobenzoate; ADC, aminodeoxychorismate; AL, ADC lyase; AS, ADC synthase; DFR, Dihydrofolate reductase; DFS, Dihydrofolate synthase; DHM, dihydromonapterin; DHN, dihydroneopterin; DHN-P, dihydroneopterin phosphate; DHNTP, dihydroneopterin triphosphate; DPS, Dihydropteroate synthase; DHNTP-PPase, DHNTP-diphosphatase; DHNA, DHN aldolase; FPGS, Folylpolyglutamate synthase; GCHI, GTP cyclohydrolase I; Glu, L-glutamate; HMDHP, hydroxylmethyldihydropterin; HPK, HMDHP pyrophosphokinase.

Folate vitamers consist of a pteridine ring attached to a *p*-aminobenzoate (*p*-ABA) group and a glutamate residue [81] (Figure 3a). Additional glutamate residues are usually attached to the γ-carboxyl group of the first glutamate residue to form a poly-γ-glutamyl tail of up to approximately eight residues. Substitutions at the N5 and N10 positions distinguish the different folate vitamers.

Folates play important roles as cofactors in reactions that involve one-carbon (1C) units in all organisms [82]. Folates are important in the synthesis of DNA and RNA, and in the synthesis of *S*-adenosylmethionine, the universal donor in methylation reactions. Folates are particularly important during periods of rapid growth such as pregnancy and fetal development [83]. Folate deficiency has been associated with the increased risk of cardiovascular diseases, anemia, and some types of cancers, as well as mild cognitive impairment, dementia and depression [83,84,85]. However, similarly to thiamin, folates may also have less-known antioxidant activities, either directly or indirectly. For instance, Nakano et al. [86] showed that 5-methyltetrahydrofolate (5-MTHF), the most active circulating form and the most dominant form of folate in most foods, delayed copper-catalyzed oxidation of low-density lipoprotein lipids to dienes in a dose-dependent manner (1–6 µM). Gliszczynska-Swiglo [87] showed that 5-MTHF and two other physiological forms, dihydrofolate (DHF) and tetrahydrofolate (THF), had antioxidant activity in the TEAC, 2,2-diphenyl-1-picrylhydrazyl (DPPH˙), and ferric acid reducing power (FRAP) assays. DHF had the highest antioxidant activities in all three assays, with values comparable or higher than those of vitamin C or α-tocopherol. Gliszczynska-Swiglo and Muzolf [88] showed that scavenging activities of folates are pH dependent. Guzman et al. [89] showed that folic acid, a non-physiological form of folate, increased levels of reduced glutathione in brain of rats with oxidative stress. Maybe more importantly, quantitative flux analyses in human cells showed that oxidation of methylene tetrahydrofolate to 10-formyltetrahydrofolate plays a major role in the reduction of NAD^+^ to NADPH with a contribution comparable to that of the oxidative pentose phosphate pathway [90]. Interactions between folates and other antioxidants also exist. For instance, Magana et al. [91] showed that vitamin C activates folate-dependent 1C metabolism [91].

Folate biosynthesis in plants, the main food source for humans, who cannot synthesize the vitamin de novo, involves three subcellular compartments (Figure 3b). In the cytosol, the sequential activities of GTP cyclohydrolase I (GCHI) [92], dihydroneopterin triphosphate (DHNTP) diphosphatase (DHNTP-PPase), and DHN aldolase (DHNA) [93] produce hydroxylmethyldihydropterin (HMDHP). DHNA also catalyzes epimerization of DHN to dihydromonapterin (DHM), which is also cleaved to HMDHP. In plastids, the sequential activities of aminodeoxychorismate (ADC) synthase (ADCS) and ADC lyase (ADCL) produce *p*-ABA from chorismate [94,95]. In mitochondria, HMDHP is pyrophosphorylated by HMDHP pyrophosphokinase (HPPK) to HMDHP-PP, which is then condensed with *p*-ABA by dihydropteroate synthase (DHPS) [96]. The glutamate residue is then added by DHF synthase (DHFS) to produce dihydrofolate (DHF) [97], which is then reduced to tetrahydrofolate (THF) by DHF reductase (DHFR) [98,99]. Finally, folylpolyglutamate synthase (FPGS) isoforms present in mitochondria, chloroplasts, and cytosol add the polyglutamyl tail [97] (Figure 3b).

The RDA for folate is 400 µg/day in adults, and 600 µg/day in pregnant women. Nutrition facts on labels of potato bags indicate that a medium size potato provides 6% of the recommended daily intake. However, folate content varies between potato genotypes. Systematic screening of potato varieties and wild relatives has shown concentrations ranging from ~200 ng g^−1^ dry weight to ~3000 ng g^−1^ dry weight [44,100,101,102], with the highest concentrations found in wild relatives. These high concentrations are about twice those found in potato varieties commonly grown in the United States, indicating genetic potential to increase folate content by breeding. Genetic engineering of the folate biosynthesis pathway is an alternative strategy that has been attempted in potato tubers with increases of up to 12-fold [103]. In addition to genetics, folate content varies during tuber development, with higher concentrations found in younger tubers [104]. Folate concentrations also increased up to 141% after 8 months of storage at 7.8 °C [77].

### 2.4. Vitamin C (L-Ascorbic Acid)

**Figure 4:**
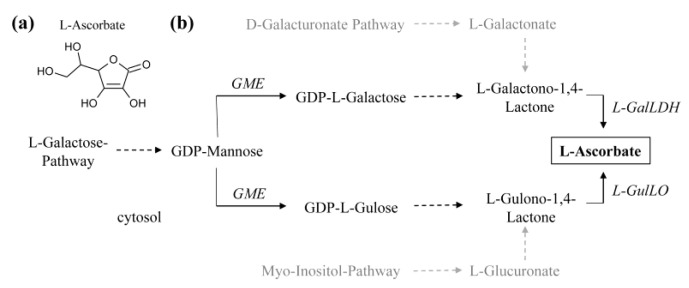
Ascorbate biosynthesis in higher plants. (**a**) Chemical structure of L-ascorbate. (**b**) Two alternative pathways, the D-galacturonate and the myo-inositol pathways, may contribute to L-ascorbate biosynthesis via L-galactonate and L-glucuronate, respectively, entering the L-galactose pathway. However, because hard proof for the existence of either pathway is missing for plants, the D-galacturonate and myo-inositol pathways are shown in light grey. L-GulLO, L-gulono-1,4-lactone oxidase; L-GalDH, L-galactose dehydrogenase; GDP-mannose epimerase (GME). Dashed arrows indicate multiple steps in between two points.

Probably one of the most classical and powerful antioxidants in plants is vitamin C (vitC) or L-ascorbic acid (Figure 4a). The molecule is widely recognized as one of the central antioxidants in living cells, and helps to detoxify a broader range of ROS such as hydrogen peroxide, hydroxy radicals, superoxide anion radicals, singlet oxygen, and peroxy radicals [105]. In humans it is also needed for a variety of biosynthetic steps such as collagen and carnitine production, amidation of certain peptide hormones and tyrosine metabolism [106,107,108,109,110]. The vitamin is essential for humans, which was historically demonstrated by scurvy disease caused by vitC deficiencies [111]. It also is critical for collagen and carnitine maintenance and cholesterol degradation to bile acid [112]. The vitamin has an estimated turn-over rate of around 1 mg kg^−1^ body per day [113,114], and requires regular supplement by food, with an RDA of 65 and 75 mg for adult (>19 years) women and men, respectively [115].

VitC functions as an antioxidant by donating electrons, thereby serving as a reducing agent. It mostly operates in plants and mammals as an enzymatic cofactor but also undergoes non-enzymatic reactions. In any case, donation of a single electron converts vitC to monodehydroascorbate (MDHA), a compound that is a resonance-stabilized radical. MDHA can either be recycled back to vitC by MDHA reductase and NADH consumption, or it can dismutate with another MDHA to generate vitC and dehydroascorbate (DHA) [116]. DHA can then be recycled back to vitC through the activity of DHA reductase and glutathione, and it also can react either with proteins and other cellular compounds (dehydroascorbylation) or be degraded to, for example, xylonic acid [117].

Potatoes are a very good source for vitC. Food labelling recommendation for vitC content by the U.S. Food and Drug Administration (FDA) for raw potatoes (143 g) is 27 mg, which represents ~30–40% of the RDA values for adult woman and men (75 and 90 mg), respectively [118]. However, the vitC content varies dependent on how the potato is processed for consumption. For example, a white fleshed potato baked with skin and flesh contains ~18 mg vitC, boiled ~18.4 mg, and microwaved ~21 mg (USDA National Nutrient Database for Standard Reference Release 28; data calculated for 143 g/sample). Although these values are not as high as some of the classical high vitC fruit resources like apple (Sugar-apples; ~51.9 mg) or orange (~84.7 mg) (USDA National Nutrient Database for Standard Reference Release 28; data calculated for 143 g/sample)), these data demonstrate that potatoes are a very good resource for the vitamin, especially for a staple food.

The data also show that the processing steps are very critical to preserve the phytonutrient value of the tuber. Not surprisingly, one can find various studies on how certain treatments affect and potentially conserve vitC content in potato including approaches such as freezing, drying, water and steam blanching, or treatment with chemicals such as sulfite [119,120,121,122,123,124,125,126]. Recent works on blanching conditions demonstrated that the temperature regime and pretreatment with citric acid can have significant beneficial impacts to preserve vitC in drying potato [119,122].

Different studies have measured great variations among potato cultivars in their vitC content, ranging from ~22 mg to up to 122 mg vitC g^−1^ dry weight [127,128]. This emphasizes the genetic potential for breeding purposes to generate high-vitC varieties, and it also shows the prospective for marketing certain potato varieties with high vitC content and their nutritional value.

Several attempts have been pursued to increase vitC content through genetic engineering [129,130,131]. In plants, the main route for vitC biosynthesis goes via the L-galactose pathway, but may also involve alternative pathways using myo-inositol or D-galacturonate as starting metabolites (Figure 4b) [129,132,133,134,135,136]. Consequently, targeting the biosynthetic pathways is the most straightforward approach for increasing vitC contents. For example, constitutive overexpression of a GDP-L-galactose phosphorylase led to a two- to three-fold increase in vitC in the tuber compared to wildtype controls [130]. The enzyme catalyzes one of the first steps in the D-glucose-6-P dependent pathway. Likewise, overexpression of a D-galacturonic acid reductase from strawberry, which converts D-galacturonate to L-galactonate, resulted in similar increases in vitC in potato (from around ~1.2 mmol vitC g^−1^ FW in wild type to 2.5 to 3 mmol vitC g^−1^ FW in transgenic plants) [137]. Interestingly, the transgenic plants also showed a more robust tolerance towards abiotic stress conditions induced by either salt (NaCl), osmotic stress (mannitol) or ROS (induced by methyl viologen) [137]. Though enhancing the myo-inositol dependent pathway via L-gulono-1,4-lactone has not been done in potato, overexpression of a gulonolactone oxidase from rats in lettuce and tobacco led to a four- to seven-fold increases in vitC [138]. Alternatively, one can also increase the content by affecting regulatory aspects of vitC metabolism, such as key transcriptional regulators, or by promoting recycling steps. Though none of these have been attempted in potato, overexpression of ERF89 in Arabidopsis and SIHZ24 in tomato, for example, resulted in significant vitC increases [139,140]. Both proteins are key transcription factors that positively regulate expression of several genes within the D-glucose-6-P dependent pathway.

Overall, potato can provide sufficient vitC to consumers on a daily basis. The crop is therefore a valuable resource for this vitamin, especially considering the vitamin’s relevance for human metabolism and health. However, there is also a good potential either through breeding efforts or genetic engineering to biofortify this important phytonutrient in the tuber.

### 2.5. Carotenoids

**Figure 5:**
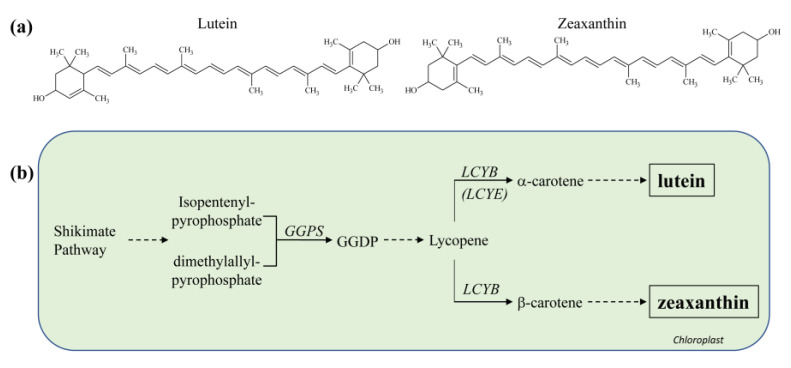
Carotenoid biosynthesis in higher plants. (**a**) Chemical structures of lutein and zeaxanthin. (**b**) Schematic drawing of some of the key steps in chloroplastidic carotenoid biosynthesis via the shikimate pathway. GGDP, geranylgeranyl pyrophosphate; GGPS, geranylgeranyl diphosphate synthase; LCYB, lycopene β-ring hydroxylase; LCYE, lycopene ε-ring hydroxylase.

Potato contains carotenoids, which help plants resist photo- and oxidative stress and influence scent and flavor [141,142]. The highest amounts are found in yellow- and orange-flesh potatoes. Over 700 different carotenoids have been identified and many are strong quenchers of singlet oxygen (^1^O_2_) and scavengers of other ROS [143]. Carotenoids are isoprenoid-based molecules synthesized in plastids with a polyene backbone consisting of conjugated C=C bonds (Figure 5a). Typically a tetraterpene skeleton is formed by linkage of the two C_20_ moieties and the resulting linear C_40_ hydrocarbon backbones are readily modified, affecting both color and antioxidant activity [144,145]. Phytoene biosynthesis is a rate-limiting step in carotenogenesis, and biosynthesis can be induced by light, photoperiod, drought and temperature [141]. Health-promoting properties of carotenoids include anti-inflammatory activity, stimulation of the immune system and decreased risk of cardiovascular disease, cancer, diabetes, anti-depressive activity and age-related macular degeneration [146,147,148,149,150,151]. The most abundant potato carotenoids, lutein and zeaxanthin, are important for eye health, and they reduce the risk of age-related macular degeneration [146,147].

Zeaxanthin is the carotenoid most responsible for orange color in potatoes, while lutein is responsible for yellow [152] (Figure 5). The Y locus encodes a β-carotene hydroxylase, which is a key determinant of tuber flesh color [153,154]. A QTL on chromosome 3 accounted for up to 71% of the carotenoid variation and is likely an allele of β-carotene hydroxylase; additional alleles affecting carotenoid amounts have been identified [155,156].

One study of Andean landraces found a range from 3–36 μg g^−1^ dry weight and up to 10 μg g^−1^ FW [157,158], while another survey of 33 Andean cultivars found up to 20 μg g^−1^ FW [159]. Some Andean cultivars contained up to 18 μg g^−1^ DW each of lutein and zeaxanthin and 2 μg g^−1^ DW of β-carotene, of which the latter is not usually present in such high amounts in commercial potatoes [160,161]. Diploid potatoes from *S. stenotomum* and *S. phureja* contained up to 20 μg carotenoids g^−1^ FW of zeaxanthin [152,162]. Total carotenoids ranged from trace amounts to 28 μg g^−1^ DW in the skin and 9 μg g^−1^ DW in the flesh in a study of 100 cultivars grown in Ireland or Spain [163,164]. Carotenoids in commercial white potatoes range from 2.7–7.4 μg g^−1^ FW, considerably less than the amounts in yellow and orange potato [165].

Transgenic approaches have greatly increased tuber carotenoid content [166,167,168]. Overexpressing three bacterial carotenoid genes in ‘Desirée’ produced a “golden potato” that had a remarkable 3600-fold increase in β-carotene to 47 μg g^−1^ DW [169], with the potential to help alleviate vitamin A deficiency in at risk populations [170]. However, a limitation of such approaches is a lack of consumer acceptance, resulting in very little consumption of transgenic potatoes in Europe or North America.

### 2.6. Vitamin E (Tocopherol)

Vitamin E (vitE) is a lipid vitamin that can integrate into membranes and in fatty acid storing oil bodies in plants. It has a major role in preventing lipid peroxidation, a chain reaction caused by ROS, which can lead to significant membrane damage in cells [171]. Therefore, the vitamin has significant health benefits for the human physiology under oxidative stress, and various studies have demonstrated that it can widely protect against lipid peroxidation in mammalian cells [172,173,174,175,176].

**Figure 6:**
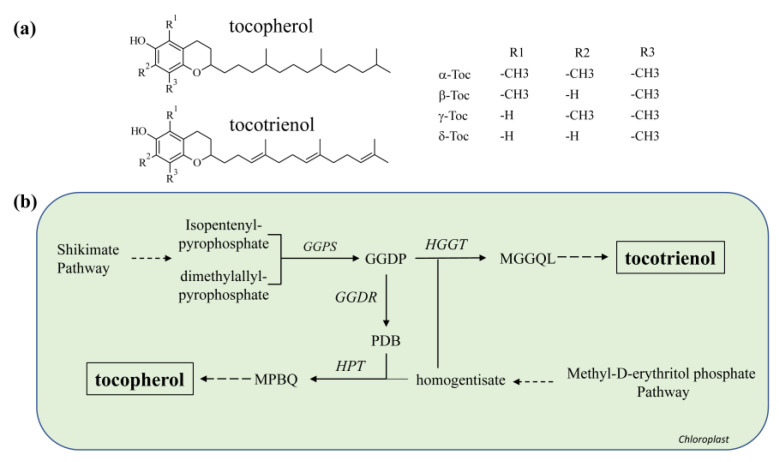
Vitamin E biosynthesis in higher plants. (**a**) Chemical structures of tocopherol and tocotrienol. (**b**) Key pathways for the biosynthesis of the vitamin include the shikimate and the methyl-D-erythritol phosphate (MEP) pathway. GGDP, geranylgeranyl diphosphate; GGDR, geranylgeranyl diphosphate reductase; GGPS, geranylgeranyl diphosphate synthase; HGGT, homogentisate geranylgeranyl transferase; HPT, homogentisate phytyl transferase; MPBQ, methylphytylbenzoquinol; MGGQL, methylgeranylgeranylbenzoquinol.

One can distinguish two major groups of vitEs in plants called tocopherol and tocotrienol, both of which have antioxidative capacities [177,178,179,180,181]. Tocopherol and tocotrienol share a chromane ring and mainly differ in their hydrophobic tails (Figure 6a). Four isomers (α to δ) exist for either vitE that differ in specific methylation patterns present at the 5, 7, and 8 carbons of the chromane ring. Tocopherols and tocotrienols also share a hydroxyl group at the 6 position (Figure 6) of the chromane ring that is required for reduction of free radicals by donation of a hydrogen atom.

VitE biosynthesis starts with the shikimate pathway leading to the production of homogentisate, which further reacts with geranylgeranyl diphosphate (GGDP) from the methylerytrithol phosphate (MEP) pathway to tocotrienol (Figure 6b). For tocopherol biosynthesis, homogentisate reacts with phytyl diphosphate, which derives either from GGDP reduction or from chlorophyll degradation (Figure 6b).

Mainly relevant for human nutrition is α-tocopherol, and the current RDA values for this compound are 15 mg per day for adult men and women [115]. In that context it is noteworthy that potato tubers preferentially store α-tocopherol over the other vitE isomers [182], and the content can highly vary among cultivars regardless of the varieties’ color [183]. Nevertheless, potato tubers do not have very high contents of the vitamin compared to other edible plants. For example, 0.07 to 0.06 mg per 100 g have been reported for raw and boiled potatoes, respectively [182], while for example broccoli and spinach have around 1.44 and 1.96 mg, respectively [182]. This is likely contributed to the fact that leaves, and specifically chloroplasts, require larger amounts of tocopherol due to photosynthetic activities that result in higher probability of single oxygen and superoxide anion generation, which can cause lipid peroxidation of thylakoid membranes [184,185].

Early attempts to increase α-tocopherol in tubers by overexpressing a *p*-hydroxyphenylpyruvate dioxygenase (HPPD) from *Arabidopsis thaliana* showed significant increases of up to 266% compared to wild type in vitE content in the tuber [186]. Similar increases (up to 258% increases in α-tocopherol compared to wild type) in vitE content were recently obtained by co-overexpressing Arabidopsis homogentisatephytyltransferase (HPT) and γ-tocopherol-methyltransferase (γ-TMT) genes in potato transgenic plants [187]. Although these increases in α-tocopherol do not reach the RDA values, they emphasize the potential of increasing vitE contents in potato. The two described approaches used constitutive overexpression constructs throughout the plant, and perhaps by using additional enzymes in the tocopherol biosynthetic pathways as well as tuber specific promoters, the levels of vitE may be further enhanced.

## 3. Phenolic Antioxidants

In addition to vitamins and carotenoids, potato contain an array of secondary metabolites that are dietarily desirable including phenylpropanoids, of which there are tens of thousands of different types. The shikimic acid pathway supplies phenylalanine from arogenate for phenylpropanoid biosynthesis. In the first committed and key regulatory step in the pathway, phenylalanine is converted into cinnamate in a deamination reaction by phenylalanine ammonia lyases (PAL), which are encoded by a multi-gene family in plants [188,189]. PALs are differentially expressed in different tissues, in response to different developmental and environmental stimuli and co-expressed with transcription factors from the ARR and LIM families. Downstream steps involve additional transcription factors including MYBs, NACs, WRKYs and bHLHs [188].

In planta, phenylpropanoids have diverse roles including mediating plant-environment interactions, plant growth and development, signal transduction, stress resistance, cell wall synthesis, flowering and pigmentation [190,191,192]. Many phenylpropanoids protect against ROS and free radicals, a trait that likely accounts for their induction by a wide range of stresses, from high light intensity to drought.

A study of 34 fruits and vegetables reported that potatoes are the third largest contributor of dietary phenylpropanoids after apples and oranges [193]. Notably, potatoes are capable of providing even higher phenylpropanoid amounts, considering that white- and yellow-flesh are by far the most consumed potato types worldwide, yet potatoes with purple- or red-flesh have markedly higher amounts.

Unlike the vitamins discussed above, phenylpropanoids do not have recommended daily allowances, yet are important for health. In addition to their anti-oxidant capacity, they have numerous health benefits that include conferring chemoprotective, anti-obesity, anti-depressive and anti-inflammatory properties, reducing the risk of metabolic syndrome, stroke and diabetes, promotion of gut, eye and cardiovascular health, longevity, and mental acuity [194,195,196,197,198,199,200,201,202,203]. The wide range of diseases phenylpropanoids are known to be effective against may reflect their efficacy against oxidative stress, given oxidative stress and inflammation underly many diseases. Dissecting the role of specific phenylpropanoids in health is a complex undertaking because they are ingested within a complex matrix resulting in cross interactions, and after ingestion are metabolized into products that can have different health-promoting properties than the parent compound [204]. Moreover, gut microbiota influence polyphenol bioefficacy and bioavailability [205]. Hundreds of trillions of microorganisms reside in the gut and the composition varies among people. Many polyphenols are relatively poorly absorbed, and thus remain in the gut for a longer time, allowing more interactions with the gut microbiota, which can biotransform the polyphenols, changing their bioavailability and efficacy; for example, into compounds active in the brain [205,206]. Polyphenols are usually present in the plant as esters, glycosides or polymers and the gut microbiota increase bioavailability by cleaving these bonds, for instance cleaving the ester linkages in conjugated hydroxycinnamates [206]. Furthermore, this is a two-way interaction, as polyphenols in turn change the gut microbiota composition. Consequently, due to the variable gut microbiome, the effect of phenylpropanoids may vary from person to person [198,207,208].

### 3.1. Phenolic Acids

**Figure 7:**
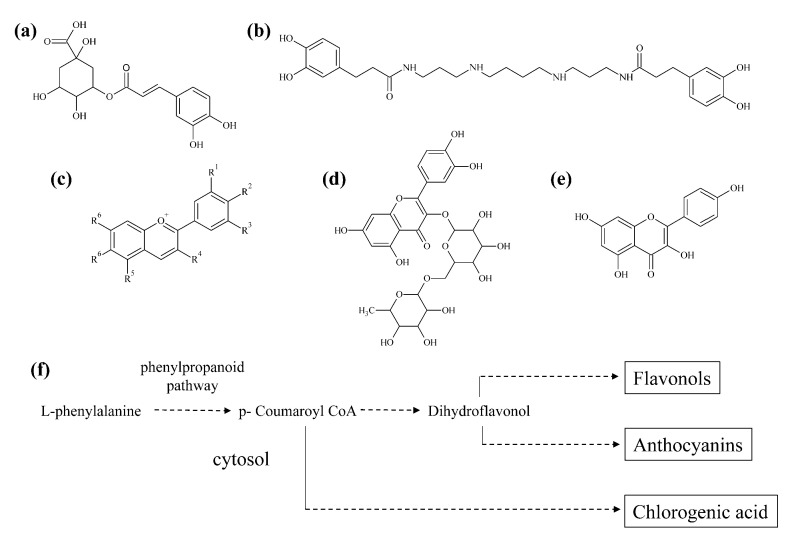
Schematic drawing of biosynthesis and chemical structures of some of the most common phenolic acids in potato. (**a**) chlorogenic acid; (**b**) kukoamine A; (**c**) general structure of an anthocyanin. The R-groups mainly represent -H, -OH, or –OCH3; (**d**) rutin; (**e**) kampferol. (**f**) Anthocyanin and flavonol biosynthesis share the phenylpropanoid pathway with each other starting with the aromatic amino acid L-phenylalanine to p-coumaroyl CoA, at which biosynthesis branches to either chlorogenic acid or flavonols and anthocyanins.

Of the five main groups of phenylpropanoids, phenolic acids and flavonoids are the two groups most relevant in potato from a dietary perspective. In white and yellow potatoes hydroxycinnamic acids are the most abundant phenylpropanoids. In white potatoes, chlorogenic acids (CGA) can constitute 90% of a tuber’s total soluble phenolics with 5-*O*-caffeoylquinic acid being the most abundant CGA [209] (Figure 7a). Multiple pathways exist for CGA biosynthesis, but the major pathway in *Solanaceae* is via hydroxycinnamoyl CoA:quinate hydroxycinnamoyl transferase [210,211]. The MYB transcription factor StAN1 mediates CGA expression, in addition to regulating anthocyanins [212].

Potatoes can contain high amounts of CGA, with purple baby potatoes shown to have over 7 mg CGA g^−1^ DW, and tubers from primitive germplasm over 12 mg/g DW [160,213]. A modest 6-ounce portion of such potatoes would provide over 250 mg of CGA, which can surpass the amounts found in a cup of coffee, which is a much better-known source of CGA [214]. Curiously, red and purple potatoes typically have much greater amounts of colorless CGA than white potatoes. However, due to the presence of anthocyanins, CGA accounts for a smaller percentage of the total phenylpropanoid content than in white potatoes [215].

CGA is readily bioavailable in humans [216,217], and is thought to have a remarkable number of health-promoting properties, including reduced risk of cancer, heart disease, strokes, Alzheimer’s and Parkinson’s [214,218,219]. CGA is also thought to be anti-hypertensive [220], and interestingly, a small human-feeding study with purple potatoes showed a hypotensive effect [221]. Tubers also contain kukoamines, small molecules with a phenylpropanoid moiety (Figure 7b). Before being found in potato, these were only reported in a Chinese medicinal plant used to treat hypertension [222]. Some nutritionists have suggested potato consumption contributes to obesity, and is a high glycemic-index food and increases the risk of diabetes. Consequently, it is interesting that CGA may decrease the risk of type two diabetes, slow the release of glucose into the bloodstream and have anti-obesity properties [223,224,225,226]. CGA had anti-obesity effects in mice, where it improved lipid profiles and decreased obesity related hormones, possibly via activation of AMP-dependent kinase [227,228]. CGA has been shown to reverse arsenic-induced brain alterations in mice and has been recommended as a natural food additive [229].

A possible impediment to developing potatoes with high levels of CGA is its potential to contribute to after-cooking darkening and bruising, although various studies report that CGA is not the rate-limiting factor in such discoloration [230,231,232].

### 3.2. Anthocyanins

Tubers also contain flavonoids, including anthocyanins (Figure 7c) and flavonols (Figure 7d,e). Roughly 20% of the total carbon flux in a plant goes through the flavonoid pathway [199]. Flavonoids have a C6-C3-C6 structure and modification of the rings with acyl, hydroxyl, methyl, and glycosyl groups gives rise to thousands of compounds. Anthocyanins are synthesized in the general flavonoid pathway, in which the first committed step converts *p*-coumaroyl-CoA and three malonyl-CoA molecules to chalcone by chalcone synthase, a type III polyketide synthase. The first committed step in the anthocyanin pathway is catalyzed by dihydroflavonol reductase (DFR). Color arises when colorless leucoanthocyanidins are converted to anthocyanidins by anthocyanidin synthase [233]. Plants contain six major anthocyanidins: cyanidin, delphinidin, malvidin, pelargonidin, peonidin and petunidin, of which all are present in potato. Numerous factors influence color, including the degree of hydroxylation/methoxylation of the B-ring. As the number of hydroxylations increase, the color becomes increasingly blue, whereas methylation can have a reddening effect [234,235]. Anthocyanins have a high antioxidant activity due to their positively charged oxygen atom but this is also influenced by the number of hydroxylations on the B-ring, with delphinidin having the most antioxidant activity and pelargonidin the least [233]. Hydroxylation may decrease anthocyanin stability, whereas methoxylation and especially acylation can increase pigment stability. A sizeable majority of potato anthocyanins are acylated with hydroxycinnamic acids, which is a desirable trait in a cooked food like potato and has led to suggestions that potato be used as a source of natural food-colorings [236,237,238].

Tuber anthocyanin in the skin is controlled by three loci, *D*, *P* and *R*, two of which are structural genes and one is an R2R3 MYB transcription factor [239,240,241]. A MYB, AN1 transcription factor complex is involved in potato anthocyanin synthesis [212,242]. Differences in the 5′ promoter region of StAN1 may be an important determinant of anthocyanin expression among genotypes [212,243]. StJAF13 is a StAN1 co-regulator, and co-expression with StAN1 or StbHLH1 increased anthocyanin amounts [244,245].

One limitation to taking full advantage of the health-benefits of high-anthocyanin red/purple/blue potatoes is that they constitute a fairly niche market, so are not as widely consumed as white or yellow potatoes. In the United States, 31% of adults older than 19 have zero daily intake of anthocyanins [234]. Anthocyanin publications in PubMed have gone from under 200 per year in 2000, to over 1000 in 2018, in part due to interest in their health-promoting effects [234]. Purple-flesh potatoes with high amounts of polyphenols and anthocyanins have shown benefits in several health studies, including induction of apoptosis, anti-cancer and anti-diabetes properties and promotion of gut health [246,247,248,249,250,251,252,253]. Anthocyanins from purple potatoes reduced alcohol-induced hepatic injury in mice [254]. A human-feeding study with purple potatoes showed markers that are indicative of reduced inflammation and DNA damage [255]. Subjects consuming purple potatoes had a significant drop in blood pressure without weight gain [221]. Males fed purple potatoes had reduced postprandial glycemia and insulinemia [256,257]. Purple potatoes provided metabolic and cardiovascular benefits to rats fed an obesity promoting diet [258] and in a human study improved arterial stiffness [259].

Anthocyanins in over 50 colored-fleshed cultivars ranged from 0.5–7 mg g^−1^ FW in the peel and up to 2 mg g^−1^ FW in the flesh [260,261]. An 11-fold variation in phenolic acids and flavanols was reported in Andean potato landraces [157,262,263] and Guincho Negra from the Andigenum group had 16 mg anthocyanins g^−1^ DW [160]. A Phureja genotype contained 41 mg g^−1^ DW [264]. In our evaluations of over a thousand potato genotypes, we observed over a 15-fold difference in phenylpropanoid amounts, including red- and purple-flesh breeding lines with over 18 mg anthocyanins g^−1^ DW. Chilean landraces had 8- and 11-fold more phenylpropanoids compared to the popular cultivars Desirée and Shepody [265].

### 3.3. Flavonols

Tubers contain modest amounts of flavonols. Unlike cultivars with high amounts of anthocyanins or phenolic acids, no cultivar is known that has high amounts of flavonols. Rather, potatoes contain small amounts of flavonols including rutin and kaempferol (Figure 7d,e). Interestingly, the low amount in tubers is not due to the lack of genes, because leaves and flowers contain very high amounts of flavonols [266].

Flavonols have a C3 hydroxyl group and a C2–C3 double bond, with the committed step in the pathway catalyzed by flavonol synthase [267]. Flavanones are converted by flavanone 3-hydroxylase into dihydroflavonols, which are precursors for both anthocyanins and flavonols [268]. Flavonols have health-promoting effects, including reduced risk of heart disease, Alzheimer dementia, asthma, obesity, emphysema, and cancer [269,270,271]. Andean potatoes contained a flavonol range of zero to 222 µg flavonol g^−1^ DW [160], while Phureja tubers contained up to 3000 µg flavonol g^−1^ DW [264]. Breeding lines containing over 430 µg flavonol g^−1^ DW are reported [215]. Wounding increases flavonols, with fresh-cut tubers having amounts up to 140 µg flavonol g^−1^ FW [272]. Light-exposed potato strips accumulated 2-fold more flavonols than those stored in the dark [272]. Both flavonols and anthocyanins are induced by cold exposure, with tubers in cold storage accumulating more flavonols [273,274]. MYB12 is a transcription factor involved in flavonol biosynthesis in Arabidopsis [275] and two MYB12s were shown to regulate potato flavonols, along with a microRNA and sucrose [266]. Overexpression of AtMYB12 in potato tubers increased flavonols over four-fold [276].

## 4. Patatin as Antioxidant

It is noteworthy that recently more and more bioactive peptides and proteins have been identified that have non-enzymatic antioxidant capabilities, and therefore can act on ROS similar to classic metabolites such as vitC or E, and which are discussed to have anti-inflammatory and anti-cancer capabilities [277,278,279,280,281].

Several works in potato have demonstrated that the main storage protein in the tuber, patatin, also has ROS scavenging abilities [282]. The 45-kDa protein can make-up more than 40% of the total soluble protein content in the tuber. Purified patatin protein is able to reduce lipoprotein peroxidation and can also efficiently quench 1,1-diphenyl-2-picrylhydrazyl (DPPH) radicals [282]. The protein is rich in cysteine and tryptophan residues, and it is discussed that these contribute to the antioxidant characteristics of patatin [282]. In fact, recent proteomic work on potato has identified patatin peptides of the sequence Phenylalanine-Tyrosine, Tyrosine-Phenylalanine-Glutamate and Proline-Proline-Histidine-Tyrosine-Phenylalanine with antioxidant activities [283].

Waste potato juice generated during starch extraction from potato is rich in other compounds, and contains around 2% protein, including patatin, with demonstrated antioxidant properties [284,285]. Therefore, waste potato juice might be a good food additive based on its antioxidant features [284,285]. Increasing patatin content may not only be beneficial for improving the ROS quenching capacities of the tuber, but also for enriching the overall amino acid content, thereby improving its general nutritional quality. However, for the processing industry this might not be of major interest because in frying processes (e.g., French fries production) certain free amino acids, such as asparagine, contribute to the generation of toxic acrylamide [286]. While patatin as an antioxidant adds another facet to the potato tuber, future work on this subject may show to what extent potato, as a whole or in context with postharvest processing steps, can be used in beneficial ways to improve nutrition.

## 5. Glycoalkaloids: Secondary Metabolites with Specific Properties

**Figure 8:**
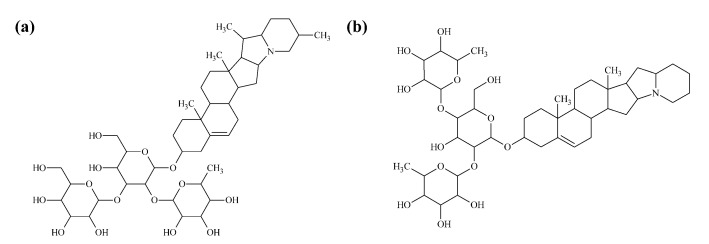
Chemical structures of two common glycoalkaloids in potato. (**a**) Solanine and (**b**) chaconine.

Solanaceous plants contain steroidal glycoalkaloids (SGAs) that can be toxic if consumed in high amounts. These alkaloids are derived from the mevalonate pathway via cholesterol with a heterocyclic nitrogen, and a C_27_ steroid conjugated to a sugar, typically a tri- or tetrasaccharide [287,288,289]. Over 80 different SGAs have been identified in potato but solanine and chaconine (Figure 8) are by far the predominant SGAs in commercial potatoes [290,291]. In many countries, including the United States, a voluntary limit of 20 mg 100 g^−1^ FW is the maximum amount allowed in a new potato cultivar. In a few European countries including Holland and Hungary, the limit is 10 mg SGAs 100 g^−1^ FW. Eating a potato with high glycoalkaloids is often first noticed as a slight burning on the tongue or throat, but at progressively higher concentrations, symptoms can include cramping, diarrhea, vomiting, rapid pulse, coma, and death [292,293]. Typically, if a commercial potato has higher amounts of glycoalkaloids it is because the potato was exposed to light and turned green, or it is an old potato that is sprouting.

SGA biosynthesis begins with the formation and condensation of C5 isoprenoid units, isopentenyl diphosphate and dimethylallyl diphosphate from acetyl-CoA, and these also serve as precursors for carotenoid biosynthesis. Three isoprenoid units are condensed to form 2-trans,6-transfarnesyl diphosphate, which is condensed to form 2,3-oxidosqualene, leading to cholesterol, which is then used for SGA synthesis [294]. High expression of HMG CoA reductase−1 (HMGR1) and squalene synthase (SQS)-encoding transcripts in potato are associated with higher SGA levels [295,296].

SGA synthesis from cholesterol involves several hydroxylation, oxidation, transamination and glycosylation steps that are becoming increasingly understood to generate SGAs [294,297,298,299,300]. The late steps of SGA biosynthesis are controlled by GLYCOALKALOID METABOLISM (GAME) genes [301,302]. Silencing GAME4 in potato reduced SGA levels up to 74-fold in both leaves and tubers [301]. Glycosyltransferases (GAME1, SGT2, and GAME2) are involved in generating the sugar moieties that combined with the steroidal aglycone moiety [303,304].

Although historically regarded strictly as toxins with no redeeming dietary merit, numerous studies over the last two decades have shown various health-promoting properties. Some SGAs are reported to have strong antioxidant activity [305]. Numerous studies have shown anti-cancer properties of SGAs, including efficacy against lung, skin, colon, liver and stomach cancer, in some cases with efficacy that approached chemotherapeutic drugs [306,307,308,309,310,311,312,313,314]. SGAs showed anti-cancer activity in a rainbow trout feeding study [315], and suppressed growth of prostate cancer cells in mice [316]. Mice treated with SGAs underwent a total remission and remained resistant to cancer cells that were subsequently injected again, which suggests these glycoalkaloids primed the immune system for long-term cancer protection [317]. Additional studies are needed to establish whether the dietary amounts of SGAs found in potato and other solanaceous plants can help protect against cancer [318,319]. SGAs potentiated the mice immune response to vaccines, suggesting SGAs can boost the immune response [320]. SGAs may also protect against infections, as Mice fed SGAs were more resistant to Salmonella infection [321]. SGAs are reported to inactivate herpes viruses [322].

Because SGAs have long been perceived strictly as “toxins” with no redeeming benefits for human health, risk-assessment studies, even recent ones, focus only on the potential toxicity, but continue to completely ignore the potential health-promoting benefits. Moreover, recent recommendations in some countries to further decrease SGA amounts in solanaceous foods that have been safely consumed for decades by billions, not only fail to consider potential health benefits, but also do not factor in the potential reduced sustainability and increased pesticide use that might result from further decreasing these natural pathogen and pest protectants in solanaceous crops.

## 6. Conclusions

Potato is one of most important crops worldwide. It has a very good calorie production and water use efficiency and represents one of the plants that has been identified by the United Nations as a crop that can help feed the growing world population in a sustainable manner. The valuable phytonutrient content of tubers, including antioxidants, show that properly prepared, potato is an excellent, healthy food resource with good nutritional properties.

We have included a table summarizing the phytonutrients discussed in this review article with their average content and suggested health benefits for humans (Table 1).

Breeding and genetic engineering approaches have demonstrated the potential (e.g., ‘golden potato’) to further improve and fortify this crop and take advantage of existing genetic diversity. It will be interesting to see how future efforts succeed in not only generating new varieties with improved traits but, and this maybe even more important, how marketing of these new varieties succeeds, especially of genetically modified potato. The latter is likely a critical bottleneck, since consumer acceptance in the end will decide whether nutritionally enhanced genetically modified potato varieties will succeed or only represent interesting research approaches that never really leave the lab. Similarly, new cultivars with higher amounts of phytonutrients generated by traditional breeding may require marketing to help inform consumers of their benefits.

## Figures and Tables

**Table 1:** Potato phytonutrients discussed in this review article, range of amounts found in tubers, and health benefits for humans.

Phytonutrient	Amount	Described Benefits	References
Vitamin B1 (thiamin)	292–2325 ng g^−1^ FW	Growth and development, proper functioning of the heart, muscles and nervous system	[22,23,24,25]
Vitamin B6 (pyridoxine)	2390 ng g^−1^ FW	anemia, neurological disorders, premenstrual syndrome, cardiovascular diseases, and cancer have been described	[63,64,65,66,67,68]
Vitamin B9 (folate)	200–3000 ng g^−1^ DW	DNA and RNA synthesis, NADPH synthesis, production of red blood cells, critical during periods of rapid growth (e.g., pregnancy, fetal development), deficiency linked to increased risk of cardiovascular diseases, anemia, some types of cancers, cognitive impairment, depression, and dementia	[83,84,85]
Vitamin C (ascorbate)	22–122 mg g^−1^ DW	Collagen and carnitine maintenance, cholesterol turn over	[112]
Carotenoids (Zeaxanthin, β-carotene)	3–36 µg g^−1^ DW	Decrease risk of cancer, diabetes, depression, macular degeneration, cardiovascular disease	[146,147,148,149,150,151]
Vitamin E (tocopherol)	700 ng g^−1^ FW	Lipid peroxidation protection	[172,173,174,175,176]
Phenylpropanoids (CGA, kukoamines, flavonols, anthocyanins)	1–41 mg g^−1^ DW	Slow release of glucose into bloodstream, promote gut health, mental acuity, decreased inflammation and risk of cancer, cardiovascular disease, obesity, diabetes, strokes, Alzheimer’s, Parkinson’s, asthma, emphysema, high blood pressure	[221,246,247,248,249,250,251,252,253,254,255,256,257,261,262,263,264,265,266,267,268,269,270,271]
Glycoalkaloids (SGA)	1–20 mg g^−1^ FW in most cultivars but can be markedly higher.	Toxic at higher concentrations. Efficacy against numerous cancers. Boost immune system. Reduced microbial infections, antiviral.	[296,297,298,299,300,301,302,303,304]
